# Encapsulation of mesenchymal stem cells in glycosaminoglycans‐chitosan polyelectrolyte microcapsules using electrospraying technique: Investigating capsule morphology and cell viability

**DOI:** 10.1002/btm2.10111

**Published:** 2018-10-01

**Authors:** Amin Vossoughi, Howard W. T. Matthew

**Affiliations:** ^1^ Dept. of Chemical Engineering and Material Science Wayne State University Detroit MI 48202

**Keywords:** chitosan, chondroitin sulfate, electrospraying, hyaluronic acid, mesenchymal stem cells, microencapsulation

## Abstract

Polyelectrolyte microcapsules are modular constructs which facilitate cell handling and assembly of cell‐based tissue constructs. In this study, an electrospray (ES) encapsulation apparatus was developed for the encapsulation of mesenchymal stem cells (MSCs). Ionic complexation between glycosaminoglycans (GAGs) and chitosan formed a polyelectrolyte complex membrane at the interface. To optimize the capsules, the effect of voltage, needle size and GAG formulation on capsule size were investigated. It was observed that by increasing the voltage and decreasing the needle size, the capsule size would decrease but at voltages above 12 kV, capsule size distribution broadened significantly which yields lower circularity. Increase in GAG viscosity resulted in larger microcapsules and cell viability exhibited no significant changes during the encapsulation procedure. These results suggest that ES is a highly efficient, and scalable approach to the encapsulation of MSCs for subsequent use in bioprinting and other modular tissue engineering or regenerative medicine applications.

## 1 INTRODUCTION

Progress in the biofabrication of implantable, engineered tissue is slowed by the challenge of assembling three‐dimensional tissue with a fully integrated microvasculature. One strategy for tissue and vessel assembly involves the fusion‐based assembly of endothelialized cell spheroids. This approach holds significant promise, but it is hampered by the need to provide substantial mechanical and organizational support to prevent uncontrolled cell aggregation and associated low vascularity. Specifically, fusion of cell spheroids is a minimally controllable cell process and can easily result in large 3D constructs or organoids with few if any functional vessels. Coating cell spheroids in an artificial basement membrane and endothelial cells prior to assembly would facilitate the transition of the surface endothelium into a postassembly, nascent vasculature. Ideally, the artificial basement membrane biomaterial would perform both as a temporary internal support for the 3D tissue and as a biologically active matrix for delivery of signaling agents and modulation of endogenous cellular responses. Microencapsulation using polyelectrolyte complexes of natural polymers is a technology that provides the necessary biological activity and may potentially approach the level of mechanical performance sought in this type of application. Microencapsulation also allows for ease of cell handling and reductions in the levels of shear stress encountered by cells during bioprinting operations and/or bioreactor cultures.

Unencapsulated cells or cell spheroids are prone to fluid shear damage in dynamic cultures and may also be subject to disruption of organization in vivo due to postimplantation migration. This can diminish both cell metabolic activity and tissue level function, reducing the efficiency of the implanted system..^1^ One approach to overcoming this obstacle is to encapsulate cells, spheroids, or organoids within biopolymer membranes, which degrade after implantation. These membranes require high permeability to nutrients, oxygen, and wastes and therefore the diffusive transport characteristics must be considered when choosing appropriate biopolymers or hydrogels for use in cell encapsulation.^2^ As a result, optimizing the parameters influencing the encapsulation process is of great importance. These parameters include the encapsulation chemistry and the biopolymers used. There are multiple methods of cell encapsulation used widely, including microfluidics‐based encapsulation, micromolding methods, and droplet/air methods. Microfluidic techniques have multiple advantages including high control over capsule size and morphology, which can generate microcapsules as small as 100 μm. However, the choice of biomaterials to be used is limited in these systems as the solutions used should have high gelation capacity such as alginate/calcium chloride or high interfacial tension such as oil/water.^3^, ^4^ Micromolding methods facilitate the formation of cell spheroids and also encapsulation of these spheroids but require specific mold designs and crosslinking methods.^5^ Air methods are easy and convenient methods of cell encapsulation, but they lack adequate control over size and uniformity of the microcapsules at the smaller size ranges.^6^ Furthermore, a general drawback for these encapsulation methods is an inability to implement mass production of microcapsules. Hence, there is a demand for an encapsulation method that can produce large quantities of capsules and is compatible with a wide range of biomaterials. Another challenge in many encapsulation techniques is the difficulty in controlling the microcapsule size and uniformity.^7^, ^8^, ^9^ We previously reported on the use of cells and cell spheroids encapsulated within glycosaminoglycan (GAG)‐chitosan polyelectrolyte membranes as a tool for tissue assembly using modular tissue engineering principles.^2^ Improvements to the technique required improvements in the droplet generation method—specifically a reduction in the droplet sizes and hence the resultant capsule sizes to enhance the nutrient transport in the fused tissue construct. Electrospraying (ES) is a method of liquid atomization using high electrical fields to overcome the surface tension of the liquid.^10^ The examples of using this technique include cell and particle encapsulation, drug delivery, nanoparticle synthesis, and film deposition.^10^, ^11^ This technique operates on the principle of an applied potential difference between two electrodes.^12^ To generate electrosprayed droplets, the polymer solution is extruded through a metal needle and the tip of the needle is maintained at a high voltage relative to the counter electrode, which can be a ring electrode through which droplets pass, or a grounded solution into which droplets are collected. These droplets are highly charged and can be in the range of nanometers to micrometers. In the ES technique, several interdependent parameters influence droplet size, size distribution, encapsulation efficiencies, and loading capacities.^13^, ^14^, ^15^ These parameters include physical properties of the liquids, voltage, needle gauge, distance to collector/counter electrode, solution flowrate, and surfactant concentration.^16^ In general, droplets formed will be in one of these modes: dripping, pulsating, cone‐jet, and multi‐jet mode. To obtain uniform droplets, the affecting parameters should be adjusted in a way that a cone‐jet is formed at the tip of the needle.^17^ In cone‐jet mode, the liquid meniscus forms an axisymmetric, uniform cone termed as the Taylor cone.^18^ As soon as the charge accumulated on the droplet overcomes the surface tension of the liquid, a uniform jet is formed. It has been seen experimentally that ES in the cone‐jet mode happens when the liquid conductivity is in the range of 10^−5^ to 10^−11^ S/m, a range within which all the semiconducting liquids fall. If the conductivity is either lower or higher than this range, droplets will be formed in dripping or multi‐jet mode, which are unfavorable with regard to uniformity.^19^ To date, the characteristics of electrosprayed droplets containing cells are not completely understood and it is important to proceed in a step‐wise manner to understand the relationship between processing parameters and characteristics of electrospray‐generated capsules before progressing to the inclusion of high value, fragile cells, and bioactive molecules.^20^

In addition to the cell type, the choice of biomaterial used for encapsulation is of great importance.^21^ Electrospray droplet generation can be adapted as a technique to encapsulate cells using the GAG‐chitosan complex method.^2^ In this research, microcapsules were formed by the complex coacervation between chitosan and GAG.^22^, ^23^ Chitosan is the second most abundant polysaccharide after cellulose.^24^ In dilute acidic solution, chitosan amino groups protonate and the polymer can subsequently form ionic complexes with a wide variety of natural or synthetic anionic species.^25^ Other specific characteristics of the chitosan include: antibacterial, antifungal, mucoadhesive, analgesic, and haemostatic properties.^26^ GAGs are also a family of highly sulfated, complex polysaccharides that play a variety of important biological roles in the body. All GAGs are negatively charged due to the presence of carboxyl and/or acidic sulphate groups. GAGs are widely distributed in animals and are essential for maintaining the integrity of the connective tissues. In solution, they are highly viscous and have low compressibility. Hence, they function as lubricating agents in articulating joints.^27^ They also bind and modulate the biological activity of many peptide growth factors and extracellular matrix proteins.^28^ Therefore, their use as components of the microcapsule structure may have beneficial effects on cell metabolic activity and functionality.^2^, ^28^, ^29^ In this study, capsule formation from ES‐generated droplets was investigated together with the viability and growth of mesenchymal stem cells (MSCs). MSCs were used as the model cell type due to their multipotency and broad use in tissue engineering systems.^29^, ^30^ Although there are many researches on using the ES technique for fabricating microparticles and microcapsules for drug delivery purposes and delivering different cells on different scaffolds, there are only a few researches concentrated on using this method as an encapsulation technique using biomaterial solutions and stem cells together.^14^, ^21^, ^31^, ^32^, ^33^, ^34^ To the best of our knowledge, this research is the first report on encapsulation of MSCs in the chitosan and GAG microenvironment using the ES technique. Capsules provide structural organization, zonation, shear protection, and scalability. In general, the novelty of this work lies in the following features. First, the ES method allows us to make uniform microcapsules at a large scale. This is one of the advantages of this technique. The only competitor to the ES method with the same capabilities are the microfluidic encapsulation methods, but as the interfacial tension of the solutions used in this research is low (below 0.1 mNm^−1^), reliable formation of microcapsules via microfluidics is extremely difficult.^35^, ^36^ Furthermore, microfluidic encapsulation using low interfacial tension materials requires additional mechanical actuators or the addition of organic liquids to stimulate phase separation or increase interfacial tension between two liquids, changes that interfere with the overall biocompatibility of the system.^37^, ^38^, ^39^ Finally, the biomaterials used for encapsulation in this research have not been previously used in the ES method. GAG‐chitosan capsules made by the ES method provide improved cell handling, structural organization, zonation, shear protection, and scalability. We propose these capsules as a modular tissue engineering platform for use as building blocks of 3D tissue structures.

## 2 RESULTS AND DISCUSSION

**Figure 1:**
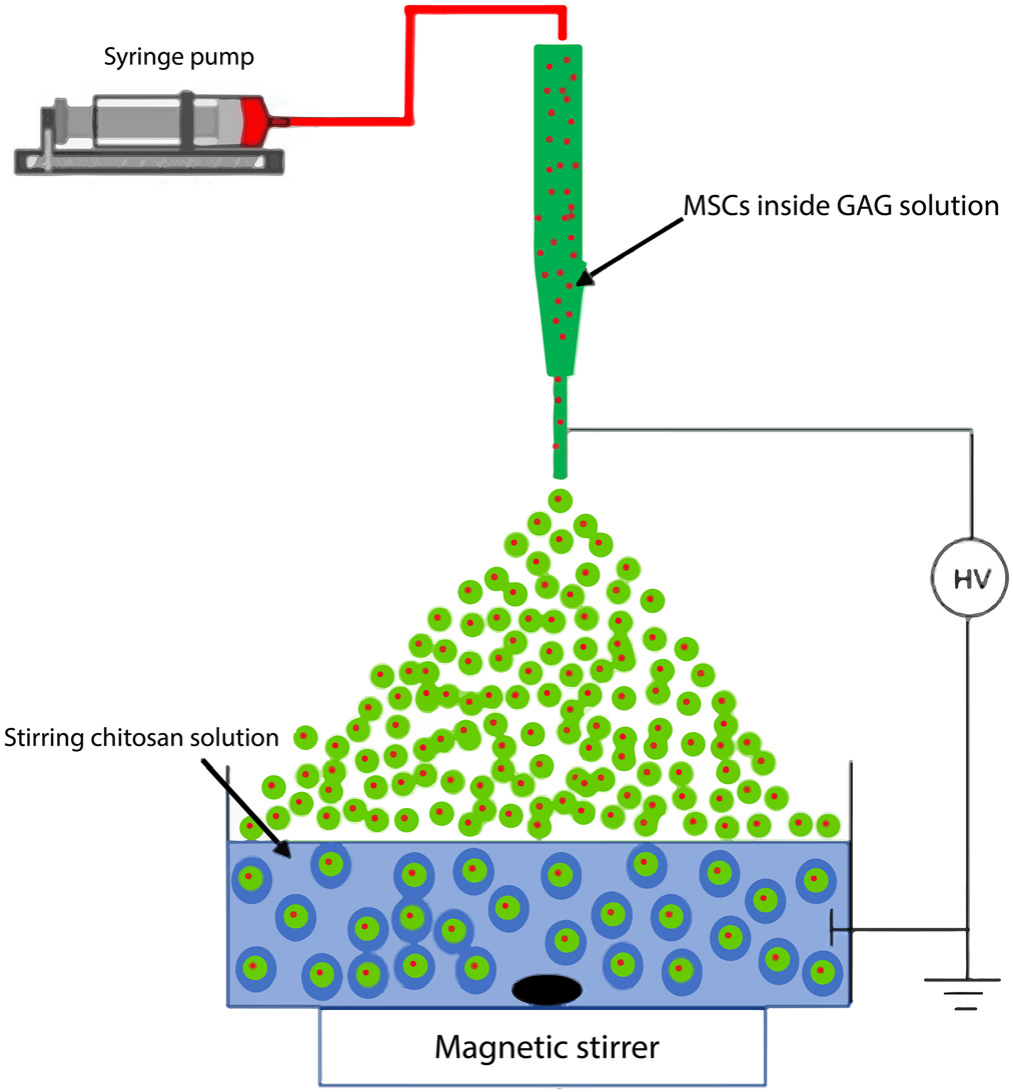
Schematic of electrospraying system

One of the main goals of this research was to make uniform, small (200‐500 μm) microcapsules using the ES technique (Figure 1). Although it has been shown that this method is able to produce uniform and monodispersed droplets, it is still a challenge to achieve this goal because of high interrelations between parameters affecting the process.^14^, ^33^ In this section, the effects of these parameters on capsule size, uniformity, shape, and cell viability were evaluated.

### 2.1 Microcapsule size evaluation

**Figure 2:**
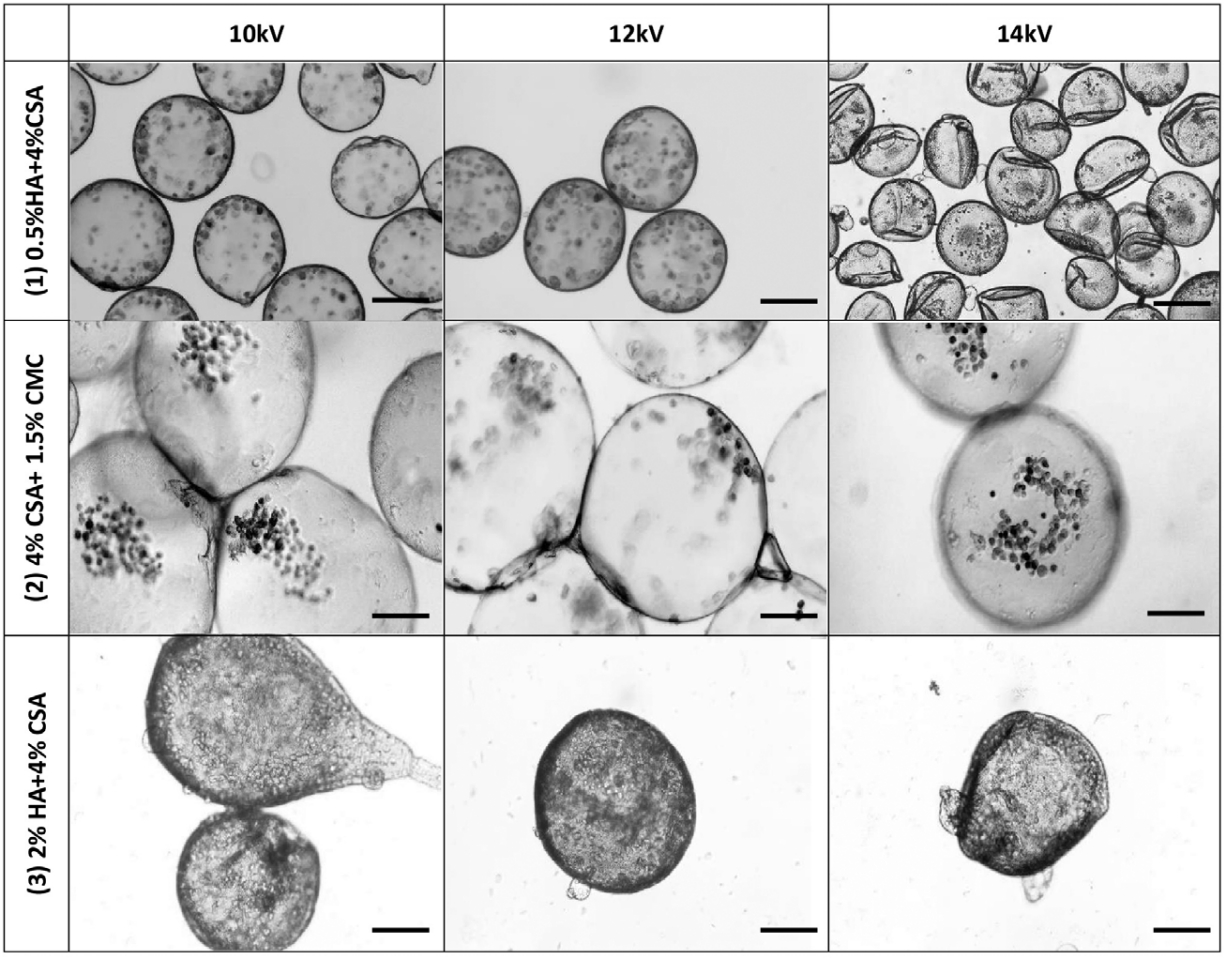
Effects of GAG formulation and voltage on microcapsule morphology with an 18 gauge needle. (1‐3): 0.5% HA + 4% CSA. (4‐6): 4% CSA + 1.5% CMC. (7‐9): 2%HA + 4%CSA, all scale bars are 200 μm

**Figure 3:**
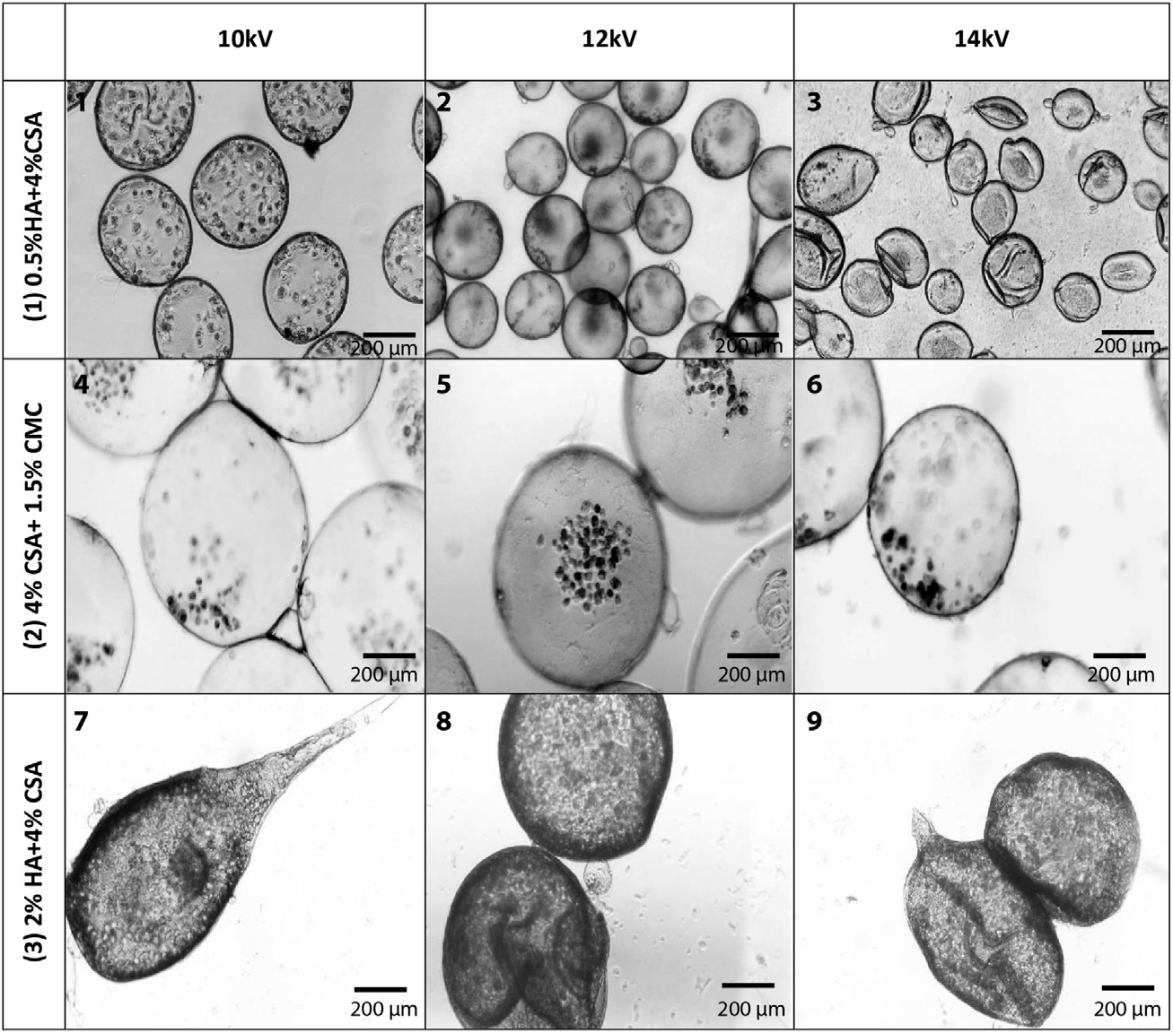
Effects of GAG formulation and voltage on microcapsule morphology with a 22 gauge needle. (1‐3): 0.5% HA + 4% CSA. (4‐6): 4% CSA + 1.5% CMC. (7‐9): 2%HA + 4%CSA

In the first set of experiments, the microcapsule size in each test was analyzed and optimized conditions were determined. It was observed that the capsule diameter was decreased by increasing the voltage from 10 to 14 kV in all three GAG types with both needle sizes. However, the reduction in capsule diameter was more obvious with the first GAG formulation (0.5% hyaluronic acid [HA] + 4% chondroitin 4‐sulfate [CSA]) compared to the two others. This was likely due to the lower viscosity of the solution when using a 0.5% HA concentration. Solutions with higher viscosity require higher voltages in order for electrostatic forces to overcome the surface tension of the liquid.^15^ Therefore, it is harder for them to detach from the tip of the needle compared to lower viscosity solutions, resulting in larger microcapsules (Figures 2 and 3).

As can be seen in Figures 2 and 3, decreasing the needle diameter decreases the average capsule size. This trend was seen in all three GAG formulations. Among the three GAG formulations, the 2% HA formulation had the highest viscosity compared to the other two. The effect of viscosity can be also seen in the lower uniformity of the microcapsules formed compared to the other formulations as the 2% HA capsules had tails due to higher viscosity and higher surface tension. The higher surface tension results in more difficult detachment of the droplets from the needle tip and results in a droplet elongation that contributes to tail formation in this GAG formulation. The higher viscosity retards the tendency of free droplets to restore a spherical shape during free fall, and the tear‐drop shape is ultimately immobilized upon formation of the capsule membrane. Capsules with the higher HA content also showed thicker walls.

**Figure 4:**
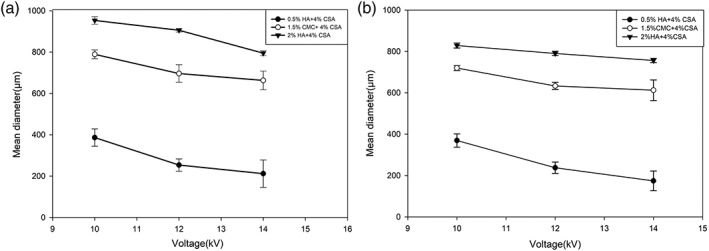
Effects of GAG formulation and voltage on microcapsule mean diameters. (a) 18‐G needle. (b) 22‐G needle. Values are means ± standard deviations from measurements on ~100 capsules. As can be seen smaller capsules were formed with 22G needle

Quantitative results for the capsule size assessment are shown in Figure 4. As shown, the decrease in the capsule size in formulation 1 is higher compared to the two others, likely due to the lower viscosity and surface tension of the solution. Moreover, by comparing the two graphs, one can clearly see that the effect of needle size on microcapsule size is less compared to those of the effects of applied voltage.

**Figure 5:**
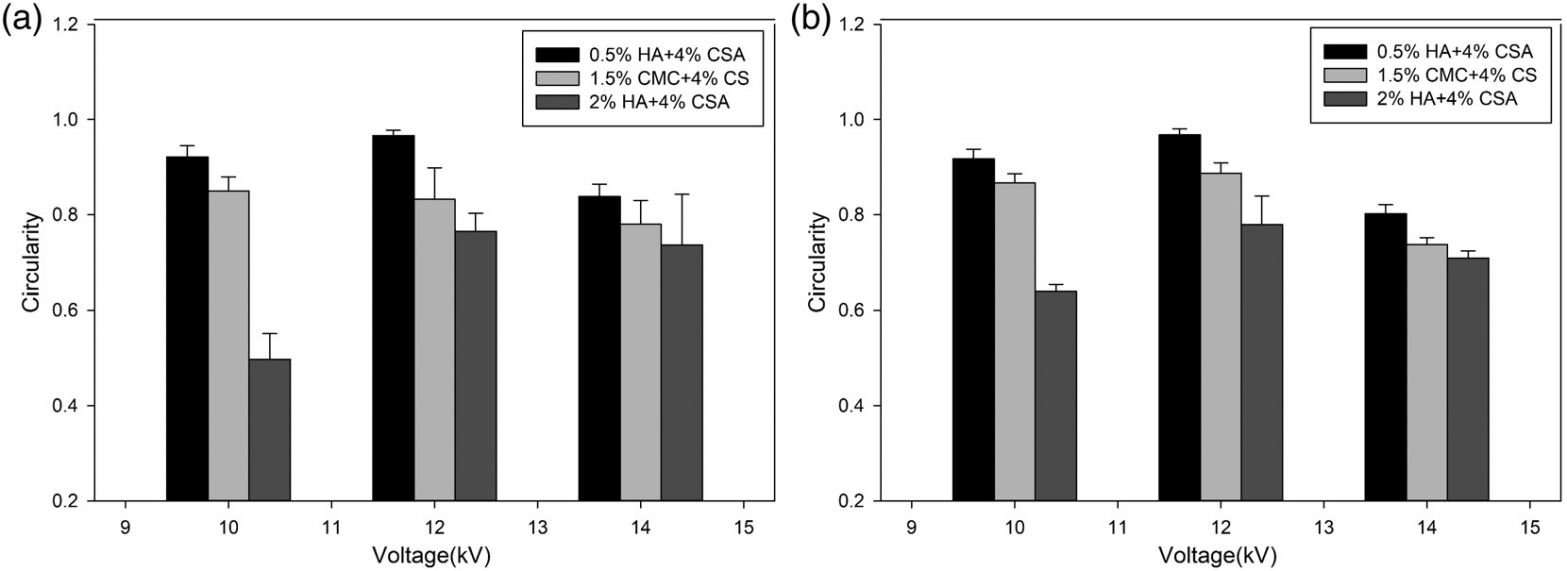
Effects of GAG formulation and voltage on microcapsule circularity. (a) Capsules formed with an 18‐G needle. (b) Capsules formed with a 22‐G needle. Values close to 1 represent perfect circles, any deviation from this value represents noncircular capsule. Values are means ± standard deviations from measurements on >100 capsules

Apart from the capsule size, the uniformity of the microcapsules is another factor that affects diffusive transport in implantation procedures.^40^ The results for the microcapsules formed by an 18 G (OD) needle are shown in Figure 5a, with similar trends being seen for the 22 G (OD) needle size in Figure 5b. These results suggest that increasing the voltage from 12 to 14 kV results in a reduction in uniformity across the whole range of microcapsules as the circularity deviates from unity. Circularity deviation from unity was also more visible for the higher viscosity formulations compared to lower viscosities. These results also suggest that using solutions with higher viscosities, in addition to yielding larger capsules, also generates less uniform microcapsules across the whole range of voltages and needle sizes. Moreover, by comparing the results for two needle sizes, it can be clearly seen that the effect of needle size on circularity of microcapsules is negligible compared to the effects of voltage and GAG formulation. Similar results have been reported in other ES studies where deviations from circularity have been reported as being related to higher voltages and higher viscosities of the electrosprayed solution.^41^ It should be noted that deviations from circularity may actually have desirable effects on encapsulated cells, as the surface to volume ratio of a given capsule increases with decreasing circularity. The net effect may be a reduction in diffusion distances within the capsules and an overall enhancement of nutrient and oxygen availability. Use of higher voltages in the ES method results in smaller microcapsules, but this has been reported to affect the cell viability and metabolic activity to a significant degree, as higher voltages can potentially inflict greater cell membrane damage leading to cell death.^32^, ^34^, ^42^ Hence, a voltage of 12 kV with a 22‐G needle size was taken as the optimized condition to achieve both smaller and more uniform microcapsules compared to the other voltages and needle size combinations. The capsules formed under these optimized conditions were later used to analyze the wall morphology and cell viability. Although not evaluated here, thermal effects at high voltages have also been reported to damage the cell wall, an effect that occurs at high voltages.^43^

### 2.2 Capsule wall morphology

**Figure 6:**
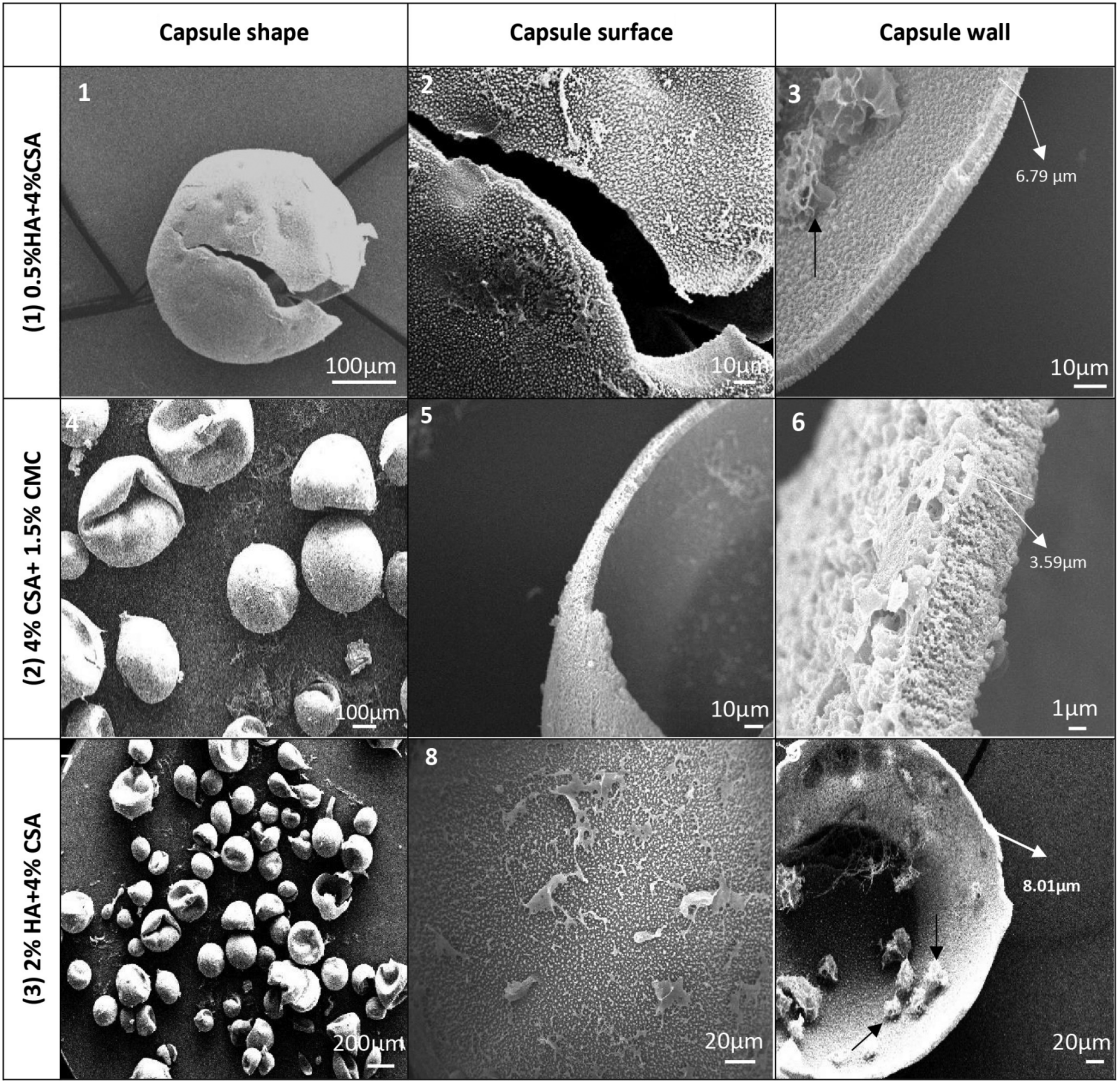
SEM images of microcapsules showing surface morphology and wall cross‐section. (1‐3) SEM images of 0.5% HA + 4% CSA capsules. (4‐6) SEM images of 1.5% CMC + 4% CSA capsules. (7‐9) SEM images of 2% HA+ 4% CSA capsules. All capsules shown were made at 12 kV using a 22‐G needle. Capsules made at 12 kV with an 18‐G needle showed membrane microstructures essentially identical to those shown here

To analyze the morphology of the microcapsule membranes in different GAG formulations, scanning electron microscopy (SEM) images were captured from the surface and interior of ruptured microcapsules. These images clearly show the wall thickness and the porous microstructure of the capsule walls (Figure 6). All microcapsules were hollow and had a porous polyelectrolyte complex membrane. The wall porosity and thickness are of importance as they directly affect the nutrient diffusion and hence the density of cells that can be maintained in a capsule of a given diameter. The porous wall structure forms by ionic complexation as the chitosan and GAGs counter diffuse in opposite directions across the interface between the two solutions. The membrane growth process is self‐limiting and usually results in cessation of thickness increase before the interior GAG solution is depleted. Excess interior GAG can subsequently diffuses out of the microcapsule during capsule washing and culture medium equilibration. SEM images also revealed that microcapsules containing HA, possessed significantly thicker membranes than those without HA. This was expected as the much higher molecular weight of the HA (~1800 kDa) would result in a more permeable capsule wall complex, ultimately allowing greater transmembrane diffusion of both chitosan and HA.

### 2.3 Cell viability analysis

**Figure 7:**
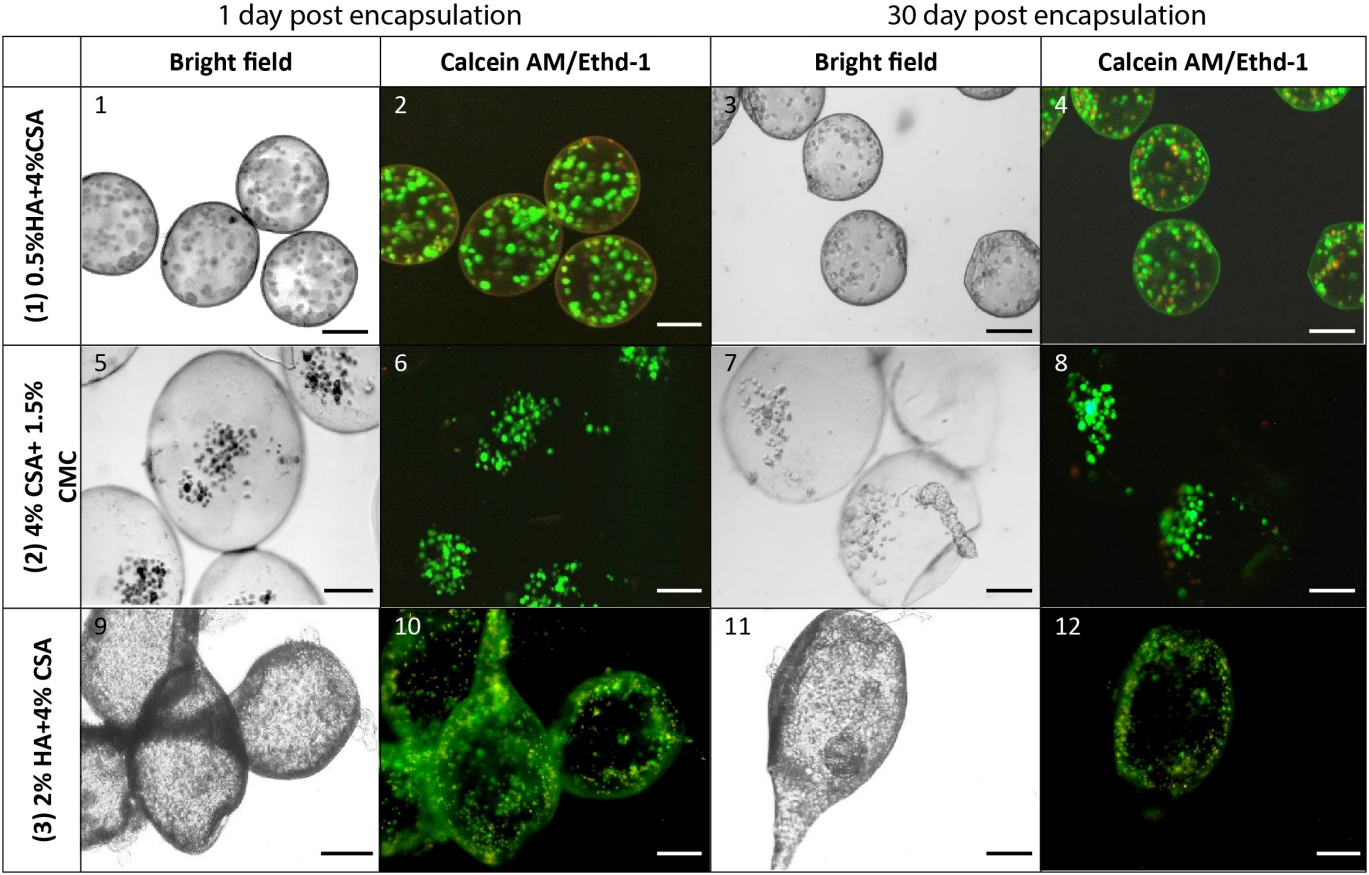
Light microscopy of encapsulated MSCs. Color images show Calcein AM (green) and Ethidium homodimer (red) fluorescence as indicators of viable and nonviable cells, respectively. Capsules shown were generated at 12 kV using a 22‐G needle. Encapsulated MSC were maintained in static culture in 6‐well plates using low glucose DMEM supplemented with 10% FBS, 50 mg/mL gentamycin, and 2.5 mg/L amphotericin B. All scale bars are 200 μm

**Figure 8:**
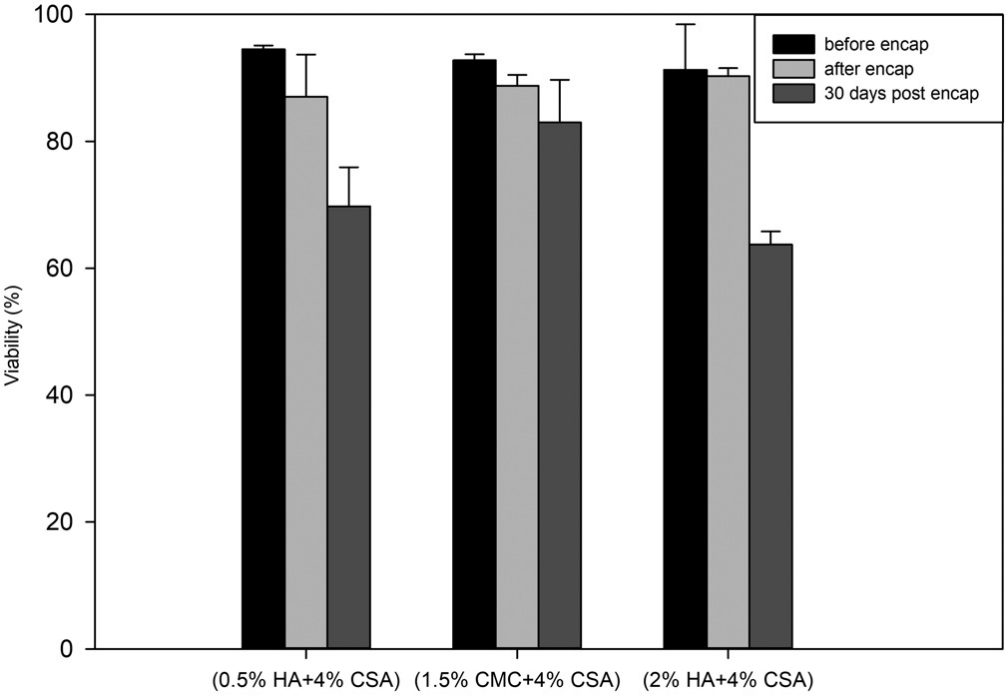
Encapsulated cell viability in the three formulations immediately before and after encapsulation, as well as 30 days post‐encapsulation. Data are mean and standard deviation from three independent culture runs. Viability percentages were obtained by counting viable and nonviable cells after fluorescence imaging of capsules exposed to the viability probes Calcein‐AM and Ethidium homodimer. All results are capsules formed at 12 kV using the 22G needle

Cell viability evaluation was performed to analyze the effect of voltage and GAG formulation. Quantitative results were extracted by counting the number of stained live and dead cells in a sample population of ~100 microcapsules. Cell viability analysis was carried out at three‐time points: before encapsulation, immediately after encapsulation, and after 30 days of static culture. All cell viability analyses were conducted on microcapsules made at 12 kV using the 22‐G (OD) needle. Figure 7 shows the Calcein AM/ETHD‐1 results for different GAG formulations. Figure 8 shows the quantitative viability results based on the Calcein AM/ETHD‐1 data. The cell viability decreases in all three formulations from Day 1 to Day 30. The decline appeared most significant for the HA‐containing formulations. However, Calcein AM fluorescence images suggested that overall cell numbers were significantly higher in individual capsules of the HA formulations as opposed to the carboxymethyl cellulose (CMC)‐based formulation. Previous studies have indicated that CMC formulations tend to exhibit higher degrees of swelling postformation (relative to initial droplet size) compared to HA formulations, which exhibit a degree of shrinkage during formation.^2^ Given this observation, we postulate that the apparently higher cell densities of the HA formulations were mainly due to disparities between formulations with regard to initial droplet size and subsequent swelling or shrinkage of capsules relative to their initial droplet sizes. Given the positive effects of smaller capsules on nutrient and oxygen transport, the HA‐based formulations would seem to have an edge, particularly when also considering the observation that encapsulated MSCs were apparently better able to attach to the interior capsule walls in this formulation, compared to the CMC based formulation where cells seemed to simply settle to the lowest point with limited obvious cell adhesion. However, the observed reductions in viability over the course of culture suggested that despite surface adhesion, the HA formulations may have lacked a currently unidentified survival signal, for example, surface‐bound integrin ligands. Additional studies to explore the modulation of the capsule environment are needed to improve the MSC survival and growth.

## 3 MATERIALS AND METHODS

### 3.1 Isolation and culture of MSCs

Chemicals and reagents used were all obtained from Sigma‐Aldrich (St. Louis, MO, USA) unless otherwise indicated. Isolation of MSCs was carried out in accordance with the recommendations of the Guide for the Care and Use of Laboratory Animals of the National Institute of Health (NIH). The MSC isolation protocol was approved by the Animal Investigation Committee of Wayne State University. In brief, MSCs were isolated from rat bone marrow by the method described by Zhang et.al^44^ and cultured in 10 cm tissue culture dishes using low glucose DMEM supplemented with 10% FBS, 50 mg/mL gentamycin, and 2.5 mg/L amphotericin B. The culture was replenished with fresh medium every 3 days. After reaching the desired confluency, cells in the third passage were trypsinized and used for ES encapsulation. All cell culture plates were kept at 37 °C in a 5% CO_2_ and 95% air humidified incubator.

### 3.2 Biopolymer materials

The materials used in the encapsulation were: chitosan (90% deacetylation) with medium molecular weight, CSA from bovine trachea (molecular weight [MW]: 50‐100 KDa), HA sodium salt (MW: 1500‐1800 KDa), CMC sodium salt (MW: 250 KDa), and polygalacturonic acid (PGA) sodium salt.

Polycation solution (chitosan) was prepared by first autoclaving 3 g of chitosan in 250 mL of water. To this suspension, 0.8 mL of glacial acetic acid was added, and the mixture was stirred for 3 to 4 hr to achieve the partial dissolution of the chitosan powder. This solution was then mixed with an autoclaved D‐sorbitol solution consisting of 19 g D‐sorbitol in 250 mL of water. After mixing, undissolved chitosan was removed by centrifugation at 500*g* for 5 min. To make the polyanion (GAG) solution, different mass concentrations of CSA, HA, and CMC were prepared in a 4‐(2‐hydroxyethyl)‐1‐piperazineethanesulfonic acid (HEPES)‐sorbitol buffer containing: 0.4 g/L KCl, 0.5 g/L NaCl, 3 g/L HEPES‐sodium salt, and 36 g/L sorbitol, adjusted to pH 7.4. A polygalacturonate (PGA, sodium salt) solution (0.1 wt % in normal saline) was used for surface stabilization of the microcapsules. Normal saline (0.9 wt.% NaCl) was used for washing the microcapsules.

### 3.3 Electrospraying system

The ES system consisted of a high voltage (HV) power supply (Gamma High Voltage research, Ormond Beach, FL, USA), magnetic stirrer, and a syringe pump for precise flow of the solution (Braintree Scientific, MA, USA), which is illustrated schematically in Figure 1. Dish cultured, rat bone marrow MSCs were trypsinized, resuspended in the GAG polyanion solution (5 × 10^6^ MSC/mL), and transferred to a 1 mL syringe. The suspension was delivered by the syringe pump to the tip of an 18 or 22 G (OD) stainless steel needle (Table 1). The needle was connected to the negative electrode of the HV system with the polycation chitosan solution (50 mL) serving as the positive electrode. The potential difference was adjusted to the desired value, and droplets generated at the needle tip were collected into 50 mL of stirred chitosan solution. In all experiment, the distance from the tip of the needle to the chitosan surface was kept at 12 mm. It was observed that for distances over 12 mm the droplets would go to the sides of the chitosan container rather than inside the solution and for lower distances the spark was formed between the tip of the needle and chitosan solution. Polyelectrolyte membrane formation around each droplet was essentially instantaneous and encapsulated the suspended cells with 100% efficiency. The microcapsules formed were washed twice with normal saline to remove excess chitosan solution, followed by a brief wash with 0.1 wt% PGA solution to achieve surface stabilization and to prevent inter‐capsule adhesion. The microcapsules were then equilibrated with culture medium for 60 min at 4°C and were then transferred to the incubator for culture or used for other analysis.

**Table 1:** Polyanion formulations, needle gauges and electrospray voltages used for MSC encapsulation. Polyanion formulations, needle gauges and electrospray voltages used for MSC encapsulation. Droplets of each polyanion solution were generated with each of the two needle sizes at each of the three electrospraying voltages listed. Droplets were collected into stirred chitosan solution to generate the encapsulating polyelectrolyte complex membrane

Needle size(G)	GAG type	Voltage (kV)
18	(1) 0.5% HA + 4% CSA	10, 12, 14
	(2) 4% CSA+ 1.5% CMC	10, 12, 14
	(3) 2% HA + 4% CSA	10, 12, 14
22	(1) 0.5% HA + 4% CSA	10, 12, 14
	(2) 4% CSA+ 1.5% CMC	10, 12, 14
	(3) 2% HA + 4% CSA	10, 12, 14

Abbreviations: GAG, glycosaminoglycans; HA, hyaluronic acid; CSA, chondroitin 4‐sulfate.

In ES, there are three parameters that mainly affect the size, shape, and uniformity of the microcapsules. An equation developed by De Shon and Carlson^45^ (Equation (1)) shows the effect of these parameters:

(1) $$r={\left[\left(\frac{3}{2\mathrm{\rho g}}\right)\left[r_{0}\gamma -2\varepsilon_{0}\left({\frac{v}{\ln\left(\frac{4H}{r_{0}}\right)}}^{2}\right)\right]\right]}^{\frac{1}{3}}$$

in which *ρ* and *γ* are the GAG density and surface tension, respectively, *V* is the applied voltage, *H* is the distance from tip of the needle to the chitosan surface, *r*_*0*_ is the internal radius of the needle, *r* is the size of the droplet detaching from tip of the needle, and ε_0_ is the air permittivity. As it can be seen from the equation, by increasing the voltage and decreasing, the needle size the size of the droplets will also decrease. Accordingly, to optimize the droplet size based on these parameters, a set of experiments were designed to monitor the effect of each parameter individually. Table 1 displays the set of experiments designed to evaluate the capsule diameter based on these parameters.

### 3.4 Microcapsule morphology and cell viability analysis

The original formulation for the encapsulation technique was developed by Matthew et al.^22^ although it was modified here using the ES technique. To investigate the effect of voltage on capsule size, three voltages 10, 12, and 14 kV were examined. Also, two needle sizes 18‐G (OD) and 22‐G (OD) were used in the experiments. Reducing the microcapsule size, while maintaining shape and uniformity in this method is important due to diffusion dependence on size and shape of microcapsules. It has been shown that uniform microcapsules in the range of 200‐500 μm show better mass transfer and diffusion properties than larger sizes.^46^ The microcapsules formed in this range were used later for cell viability tests.

For SEM imaging, microcapsules were fixed in 2% glutaraldehyde in a cacodylate buffer, washed with water, dehydrated in ethanol, and flash frozen in liquid nitrogen. The frozen mass was lyophilized until dry and then sputter coated with gold for SEM imaging at an acceleration voltage of 15 kV on a JEOL JSM‐7600 microscope. In addition to size comparisons, the shape of the microcapsules was analyzed as an indicator of the capsule uniformity. Here, the circularity (Equation (2)) was used as a tool to evaluate the uniformity of microcapsules formed in each voltage with the corresponding needle size and GAG formulation. “*A”* and “*P”* represent area and perimeter of the microcapsules, respectively. Values equal to 1 represent a perfect circle while smaller values suggest deviations from circularity, and a higher surface‐to‐volume ratio of the microcapsules. All results were calculated for a minimum of 100 capsules in each experiment.

(2) $$f=\frac{4\mathrm{\pi A}}{P^{2}}$$

Cell viability was investigated using Calcein‐AM/ethidium homodimer method (Cytotoxity kit L3224; Invitrogen). As the microcapsule wall acts as a diffusion barrier, high concentrations of the dyes were used. In brief, 4 μL of Calcein‐AM stock and 4 μL of ethidium homodimer stock solutions were added to 1 mL of PBS and the solution was mixed thoroughly. About 600 μL of the prepared dye solution was added to each well of a 12 well plate containing the microcapsules. The microcapsules were then incubated for 30 min and washed afterward with PBS to remove the background dye. Microcapsules were then imaged under a fluorescence microscope (Nikon Diaphot 300) and the number of dead and live cells in a sample population of ~100 microcapsules were counted.

## 4 CONCLUSIONS

In this study, the effects of electrospray droplet generation on characteristics of GAG‐chitosan microcapsules and encapsulated MSCs were investigated. The ES technique yielded fairly uniform microcapsules at sizes that were typically subject to large size dispersion using the traditional air atomization methods. Higher voltages produced smaller capsules with a narrow size distribution, but also resulted in less spherical capsule shapes. Solution viscosity was determined to be an important variable in that smaller, more circular capsules could be generated easier using lower solution viscosities than by either increasing voltages or reducing needle diameters. HA‐based capsules were found to support greater cell adhesion to capsule walls than the carboxymethyl cellulose‐based formulation. These results demonstrated that electrospray droplet generation is a viable and superior alternative to previous air flow methods for encapsulating MSCs within glycosaminoglycan‐chitosan polyelectrolyte membranes.

## CONFLICT OF INTEREST

The authors have no conflicts of interest to declare.
